# Bacterial co-occurrence with pulmonary TB, a respiratory tract infection (RTI): A cross-sectional study in a resource-limited setting

**DOI:** 10.1016/j.jctube.2025.100534

**Published:** 2025-05-10

**Authors:** Mpho Magwalivha, Mpumelelo Casper Rikhotso, Leonard Owino Kachienga, Rendani Musoliwa, Ntshunxeko Thelma Banda, Maphepele Sara Mashilo, Thembani Tshiteme, Avheani Marry Mphaphuli, Hafsa Ali Mahamud, Sana Patel, Jean-Pierre Kabue Ngandu, Sana Patel, Natasha Potgieter, Afsatou Ndama Traoré

**Affiliations:** Department of Biochemistry and Microbiology, Faculty of Sciences, Engineering & Agriculture, University of Venda, Thohoyandou 0950, South Africa

**Keywords:** Co-infection, HIV, *Mycobacterium tuberculosis*, Resistance

## Abstract

**Background:**

Bacterial co-infections significantly affect the treatment outcomes of tuberculosis (TB) patients, particularly in resource-limited settings. Misdiagnosis of TB co-infections accelerate disease progression and contribute to the development of drug resistance, leading to higher mortality and morbidity rates, especially in underserved areas. This study aimed to investigate bacterial co-infections in patients with pulmonary tuberculosis in a rural Vhembe region of Limpopo, South Africa.

**Materials and methods:**

A total of 100 sputum together with 100 blood samples were collected from TB patients who were undergoing TB treatment. DNA isolates were used as templates for PCR using the Anyplex™MTB/NTMe Assay kit, and subsequently, the Allplex™ MTB/MDR/XDRe Assay kit was used for the multiple detections of *Mycobacterium tuberculosis* (MTB) and resistance to first line and second line anti-TB drugs. Co-infections were determined using the Allplex™ Bacteria(I) & (II) Assay kit. HIV status of patients was determined using blood testing kits.

**Results:**

Majority of study participants were male (55 %) and aged between 36 and 55 (54 %), while female were 46 % of the population. Bacterial species detected included non-tuberculous mycobacteria (NTM) in 67 % of participants, Aeromonas spp. (19 %), Vibrio spp. (2 %), and E. coli (2 %). Multidrug-resistant *Mycobacterium tuberculosis* (MTB) strains were identified in 2 % of the cohort. There was a significant association between employment status and age (p = 0.00), as well as between HIV status and age (p = 0.03). While no significant associations were found between HIV status and the presence of NTM or other bacterial co-infections (p = 0.19 and 0.21, respectively), the majority of Aeromonas spp. and NTM cases were observed among HIV-positive participants. Notably, 36 of the NTM cases occurred in individuals living with HIV.

**Conclusion:**

The study findings suggest that age, socioeconomic status, and gender play a role in the development of TB, HIV, and other bacterial infections, which could further complicate treatment outcomes in patients. These factors likely contribute to increased vulnerability to co-infections, emphasizing the complex interplay between TB and HIV in these populations. Additionally, the study emphasises the importance of considering these socio-demographic factors in public health interventions to reduce the burden of TB-HIV co-infection and associated bacterial infections.

## 1 Introduction

Tuberculosis is one of the leading causes of infectious illness and mortality worldwide [1,2,3]. 10.6 million people fell ill in 2021, an increase (4.5 %) compared to 2020 TB cases, with about 86 % falling under the WHO regions [4]. South Africa ranked number 6 among 22 TB high-burden countries with an estimated rate of 860/ 100 000 population in 2014 [5,6]. The rate has been shown to decrease (615/ 100 000 population) in 2020 statistics; however, it is exceptionally high compared to the global rate (130/ 100 000 population*)* [5].

Over the years, MTB has developed a drug-resistant strain, resulting in a public health issue [7,8]. Co-infection of pathogens that may facilitate the pathogenesis of TB has been a problem for years, contributing to the failure of TB control globally [9]. The leading co-infecting pathogen in TB cases is HIV, and nearly one in every four deaths among people living with HIV is attributed to TB [10,3]. HIV is proven to be a potent risk factor for TB and complicates every aspect of TB care, starting from prevention to diagnosis and treatment. It has been reported that TB increases the progression of HIV and contributes to slower CD4 recovery [11]. Moreover, bacterial infections are evident in TB patients, and the simultaneous occurrence of infectious microbes has been suggested to lead to delayed diagnosis or even misdiagnosis, resulting in inadequate treatment [12,13,14,15]. Hence, it is important to consider underlying co-infections during TB diagnosis tests, especially in patients with drug-resistant TB or non-tuberculosis mycobacteria (NTM).

Several studies have reported on co-infection pathogens such as SARS-COV2, which lowers the ability of the immune system to respond to infections [16,17,18,19]. Other lower respiratory tract infections (LTRIs) pathogens include *Streptococcus pneumoniae, Staphylococcus aureus,* Influenza*,* and rhinoviruses. Typically, LTRIs affect the host's lungs, mainly the bronchi and trachea, leaving the host with symptoms such as coughing, shortness of breath, fever and malaise [20]. Aeromonas species are included in pathogens usually isolated from respiratory samples and associated with various human infections [21,22]. *Escherichia coli (E. coli)* has been reported to cause extraintestinal illnesses in humans, including pneumonia and respiratory infections [23].

South Africa has one of the worst tuberculosis epidemics in the world, with high disease burden, incident rates, HIV co-infection rates and growing epidemics of multidrug-resistant TB [24,25]. While there is substantial data on studies focusing on HIV/AIDS co-infection worldwide, such studies are scarce, particularly in the Limpopo province of South Africa, a resource-limited setting. Few studies have focused mainly on the prevalence and associated risk factors of TB around the communities as well as HIV/TB co-infection. Thus, this highlights the limitation of studies focusing on the co-infections of other pathogens in TB patients around the Vhembe area. Therefore, this study aimed to determine the bacterial co-infection on active TB patients in the Vhembe district (Limpopo), assessing the burden of the disease and highlighting the risks posed by the co-infections.

## 2 Materials & methods

### 2.1 Ethical consideration

The study proposal was submitted and ethically reviewed by the University of Venda Research Ethics Committee (UREC) and assigned an ethical clearance number (SMNS/20/MBY/13/2104). The provincial ethical consideration was obtained from the Limpopo Provincial Department of Health (LP_2021-11–001), and permission to access healthcare facilities was obtained from the Vhembe district Department of Health. Selected healthcare facilities were visited to introduce the study and seek permission to conduct the study in their facilities. A written consent was obtained from all participants. The participants' rights were considered, and they were allowed to withdraw without prejudice.

### 2.2 Design and setting

**Fig. 1:**
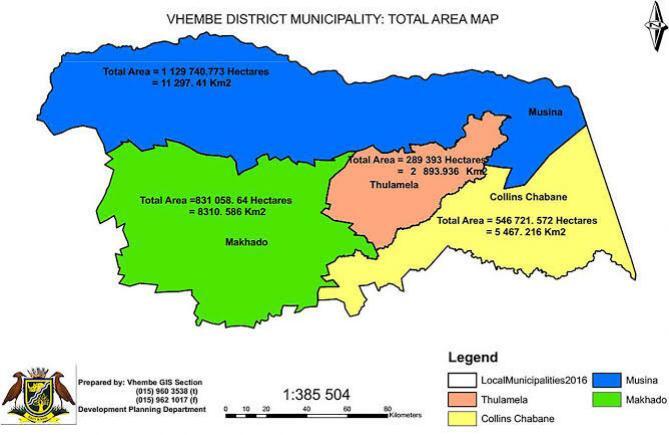
Vhembe district municipality located in Limpopo province of South Africa(Vhembe district municipality profile, 2019).

The study was conducted in the Vhembe region, Limpopo, South Africa. Fig. 1 shows a map of Vhembe district municipalities with all local municipalities (Makhado, Thulamela, Musina and Collins Chabane), which have health facilities to a total number of 116 clinics, 6 district hospitals and one regional hospital (Vhembe district municipality profile, 2019).

### 2.3 Sampling

The study was conducted over a period of nine months between 2022 and 2023 in various healthcare facilities in the study area. A total of 100 enrolled participants were above the age of 18 and were on anti-TB treatment. Most participants (94 %) were outpatients recruited from the clinics during their check-ups, while 6 % were hospitalised patients. Furthermore, the participants visited the healthcare facilities after experiencing TB symptoms. A brief study background was explained to the participants, and a consent form was issued. A detailed structured questionnaire was used to obtain information about the patient's demographics and medical conditions (co-morbidities). A professional nurse assisted with collecting blood and sputum samples from the participants. Samples were then stored in a cooler box with ice until transported to the University of Venda Microbiology TB unit laboratory for further analysis.

### 2.4 Laboratory analysis

#### 2.4.1 HIV test

The HIV status of patients was determined using a rapid U-Test HIV/AIDS kit, following the manufacturer's instructions (Humor Diagnostica, Hermanstad, SA). This was done to determine HIV co-infection among TB patients.

#### 2.4.2 Sputum pre-treatment

Samples were removed from the cooler bags and left at room temperature for about 15 min. Pre-treatment was done: A total of 1.5 ml sputum sample already mixed with 4 % NaOH (Sigma-Aldrich, US) was transferred to 1.5 ml sterile tubes and centrifuged at 15000 x g (13 000 rpm) for 5 min. The supernatant was discarded from each tube by pipette. About 1 ml of 1X PBS (Merck, Germany) solution was added and mixed well. The tubes were centrifuged at 15 000 x g (13 000 rpm) for 5 min. This step was repeated twice. The pellet was kept for DNA extraction.

#### 2.4.3 DNA extraction

A 100 µl of DNA Extraction Allplex™ kit (Seegene, Seoul, South Korea) solution was added into the 1.5 ml tubes with pellet, and 10 µl of internal control was added into the mixture. Tubes were briefly vortexed and placed into a centrifuge for 15,000 x g (13,000 rpm) for 5 min. After centrifugation, tubes were placed into a heating block at 100˚ C for 20 min. After boiling, the samples were centrifuged at 15,000 x g (13,000 rpm) for 5 min. 5 μl of supernatant was stored as a PCR template at −20 °C for further analysis (mPCR).

#### 2.4.4 MTB molecular detection

Anyplex™ MTB/NTM (Seegene Korea, Seoul) Real-time detection was used to analyse the DNA extracted from sputum samples to detect targeted sequences of MTB/NTM. The Allplex™ kit can detect up to 49 NTM species, but it does not differentiate between specific species—only indicating a general NTM-positive result. A total of 15 µl master mix was done by mixing 2 μl 10X MTB/NTM OM, 3 μl RNase-free Water and 10 μl 2X Anyplex™ PCR Master Mix (with UDG). The master mix was briefly vortexed, followed by centrifugation. A 20 µl PCR reaction volume containing 15 μl of PCR Master mix and 5 μl of each sample's nucleic acid was added into 0.2 ml PCR tubes. The mPCR was carried out according to the manufacturer's protocol. Gel electrophoresis was done to analyse the sputum DNA fragment. All the positive MTB-tested samples were subsequently subjected to Allplex™ MTB/MDR/XDRe (Seegene, Seoul, South Korea) for the multiple detection of MTB/MDR/XDRe.

#### 2.4.5 Co-infections

To detect co-infection pathogens in the extracted sputum DNA, Allplex^TM^ Bacteria (I) & (II) Assay kits (Seegene, Seoul, South Korea) were used. The PCR master mix containing 5 µl of 5 X GI-B(I) MOM, 10 µl of RNase-free water, and 5 µl of EM2 was prepared in a 0.2 ml PCR tube. A total of 5 µl DNA was added into the PCR master mix to make up a total volume of 25 µl PCR reaction and placed in a CF96 TM Real-time PCR System (Bio-Rad, Hercules, CA, USA) for amplification. The thermal conditions for successful amplification were set as follows: 50 °C for 20 min followed by 95 °C for 15 min and 45 cycles of 95 °C for 10 s, 60 °C for 1 min, and 72 °C for 30 s (where fluorescence was detected at 60 °C and 72 °C).

### 2.5 Statistical analysis

The data was recorded on an Excel spreadsheet. A descriptive statistical analysis was performed on TB patients. A chi-square test was conducted using IBM SPSS Statistics (Version X, IBM Corp, 2021) to explore the association between gender, age, various factors, and HIV status association to bacterial co-occurrence. The p-values were generated to measure significance levels among the categorical data in the current study.

## 3 Results

This study aimed to investigate bacterial co-infections in patients with pulmonary tuberculosis in a rural Vhembe region of Limpopo, South Africa. As presented in Table 1, most participants in the current study were male (55 %) and between the ages of 36 and 55 years (54 %). The most common group of female participants were aged between 18–35, comprising 55.17 %, while the most common male participants were in the middle-aged (35–55) and elderly (56–80) categories, accounting for 55.56 % and 58.82 %, respectively. Furthermore, most participants had attained secondary education (grades 8–12), with 56 % reporting this level of education. However, there was no significant association (*p =* 0.84) between gender distribution and the education level participants attained. When it comes to employment status, female participants had the highest proportion of unemployment (53.57 %), while male participants were more likely to be employed (63.16 %) and self-employed (70.00 %). However, this also did not reach statistical significance (p-value = 0.84). HIV prevalence was notably high, affecting 61 % of participants, with males being disproportionately affected (55.74 %). Despite the notable difference, the association was not statistically significant (p-value = 0.85).

**Table 1:** Epidemiologic and Clinical Characteristics of the study cohort based on gender.

**Characteristics**	**Category**	**Gender** **Female (N = 45) Male (N = 55)**		**Total (N = 100)**	***P-*value**
**Age**	18–35	16 (55.17)	13 (44 83)	29 (29.00)	0.24
	36–55	24 (44.44)	30 (55.56)	54 (54.00)	
	56–80	5 (29.41)	12 (70.59)	17 (17.00)	
**Education**	Grade1-7	7 (41.18)	10 (58.82)	17 (17.00)	0.84
	Grade 8–12	26 (46.43)	30 (53.57)	56 (56.00)	
	No education	3 (33.33)	6 (66.67)	9 (9.00)	
	Tertiary	9 (50.00)	9 (50.00)	18 (18.00)	
**Employment status**	Employed	7 (36.84)	12 (63.16)	19 (19.00)	0.26
	Self-employed	6 (30.00)	14 (70.00)	20 (20.00)	
	Student	2 (40.00)	3 (60.00)	5 (5.00)	
	Unemployed	30 (53.57)	26 (46.43)	56 (56.00)	
**HIV status**	Negative	18 (46.15)	21 (53.85)	39 (39.00)	0.85
	Positive	27 (44.26)	34 (55.74)	61 (61.00)	
**NTM/MTB**	MTB	9 (50.00)	9 (50.00)	18 (18.00)	0.45
	MTB + NTM	8 (66.67)	4 (33.33)	12 (12.00)	
	NTM	23 (41.18)	32 (58.18)	55 (55.00)	
	ND	6 (40.00)	9 (60.00)	15 (15.00)	
**Drug resistance**	Resistance strains	1 (50.00)	1 (50.00)	2 (2.00)	0.55
	N/A	29 (41.43)	41 (58.57)	70 (70.00)	
	No	15 (53.57)	13 (46.43)	28 (28.00)	
**Co-occurrence**	Aeromonas (Aer)	6 (35.29)	11 (64.71)	17 (17.00)	0.85
	Aer and Vibrio	1 (50.00)	1 (50.00)	2 (2.00)	
	E. coli	1 (50.00)	1 (50.00)	2 (2.00)	
	No	37 (46.83)	42 (53.16))	79 (79.00)	
**Total**		45 (100.00)	55 (100.00)	100 (100.00)	

Aer = Aeromonas spp.

The total cases of NTM spp detected in the study were 67 (67 %), and co-occurrence of MTB and NTM was observed in 12 % of patients, with a higher prevalence in females (66.67 %) (Table 1). However, NTM cases were more prevalent in males (58.18 %) with no statistically significant difference (p-value = 0.45) in detecting Mycobacterium between genders. Other detected bacteria, namely, *Aeromonas* spp.*, Vibrio* spp.*, and E. coli,* were detected in the study population (19 %, 2 %, and 2 %, respectively) in the study population. Aeromonas was commonly detected in male participants (64.71 %), whereas *Vibrio pp.* and *E. coli* were detected in the same samples of both genders. However, no statistically significant difference was found in the simultaneous presence of these bacteria between genders in TB patients (p-value = 0.85). Additionally, both male and female participants had one case each of drug-resistant strains, with no significant difference (p-value = 0.55) between genders (Table 1).

The distribution of employment status across different age groups (18–35, 36–55, and 56–80) revealed a statistically significant difference (p-value = 0.00), suggesting a strong association between employment status and age (Table 2). Among participants aged 36–55, a higher proportion were employed (68.42 % out of 19) and self-employed (75.00 % out of 20). A high proportion of unemployment was also observed in the 36–55 age group, with 48.15 % out of 56 individuals remaining unemployed. A statistically significant difference (p-value < 0.00) was observed between age groups and education status. Participants aged 36–55 had the highest proportion of secondary education (62.50 %), while those aged 18–35 had the highest level of tertiary education (34.48 %, 100 % out of 9 participants) (Table 2). The 36–55 age group also had the highest proportion of HIV-positive cases (62.30 %), with HIV status significantly associated with age (p-value = 0.03). In contrast, the 18–35 age group had the highest proportion of HIV-negative individuals (58.62 %) (Table 2). There were no statistically significant differences between age groups concerning bacterial co-occurrence and drug resistance cases, with p-values of 0.41 for both (Table 2).

**Table 2:** Characteristics of the study cohort distributed by age.

**Characteristics**	**Category**	**18**–**35**	**Age** **36**–**55**	**56**–**80**	**Total**	**P-value**
Employment status	Employed	5 (26.32)	13 (68.42)	1 (5.26)	19 (19.00)	0.00
	Self-employed	3 (15.00)	15 (75.00)	2 (10.00)	20 (20.00)	
	Student	5 (100.00)	0 (0.00)	0 (0.00)	5 (5.00)	
	Unemployed	16 (28.57)	26 (46.43)	14 (20.00)	56 (56.00)	
Education	Grade1-7	2 (11.76)	7 (41.18)	8 (47.06)	17 (17.00)	<0.00
	Grade8-12	17 (30.36)	35 (62.50)	4 (7.14)	56 (56.00)	
	No education	0 (0.00)	4 (4.44)	5 (55.56)	9 (9.00)	
	Tertiary	10 (55.56)	8 (44.44)	0 (0.00)	18 (18.00)	
HIV status	Negative	17 (43.59)	16 (41.03)	6 (15.38)	39 (39.00)	0.03
	Positive	12 (19.67)	38 (62.30)	11 (18.03)	61 (61.00)	
NTM/MTB	MTB	6 (33.33)	11 (61.11)	1 (5.56)	18 (18.00)	0.11
	MTB + NTM	1 (1.67)	10 (83.33)	1 (8.33)	12 (12.00)	
	ND	2 (13.33)	9 (60.00)	4 (26.67)	15 (15.00)	
	NTM	20 (36.36)	24 (43.64)	11 (20.00)	55 (55.00)	
Co-occurrence	Aer	7 (41.18)	9 (52.94)	1 (5.88)	17 (17.00)	0.41
	Aer, Vibrio	0 (0.00)	2 (100.00)	0 (0.00)	2 (2.00)	
	E. coli	0 (0.00)	2 (100.00)	0 (0.00)	2 (2.00)	
	No	22 (27.85)	41 (51.90)	16 (20.25)	79 (79.00)	
Drug resistance	Resistance	0 (0.00)	2 (100.00)	0 (0.00)	2 (2.00)	0.42
	N/A	22 (31.43)	33 (47.14)	15 (21.43)	70 (70.00)	
	No	7 (25.00)	19 (67.86)	2 (7.14)	28 (28.00)	
Total		29 (100.00)	54 (100.00)	17 (100.00)	100 (100.00)	

As presented in Fig. 2A, Fig. 2B, the analysis of participants with HIV and their association with NTM and other bacterial co-occurrences did not reveal any significant associations, with p-values of 0.19 and 0.21, respectively. However, the overall prevalence of *Aeromonas spp*. was seen in HIV-positive participants. Similarly, the majority prevalence of NTMs (36 cases) was seen in HIV-positive participants.

**Fig. 2A:**
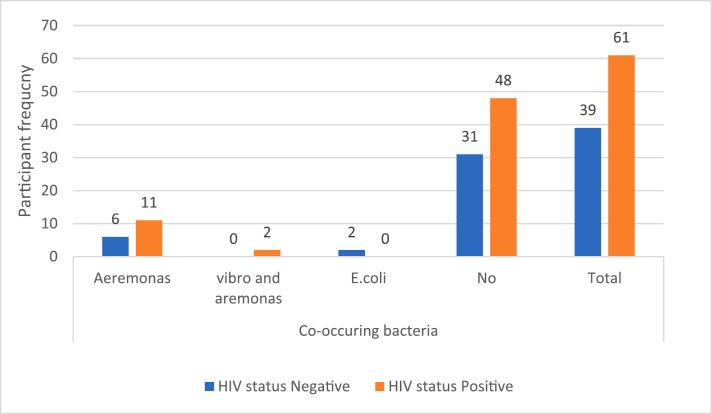
TB-HIV co-infection associated with co-occurrence of bacterial spp.

**Fig. 2B:**
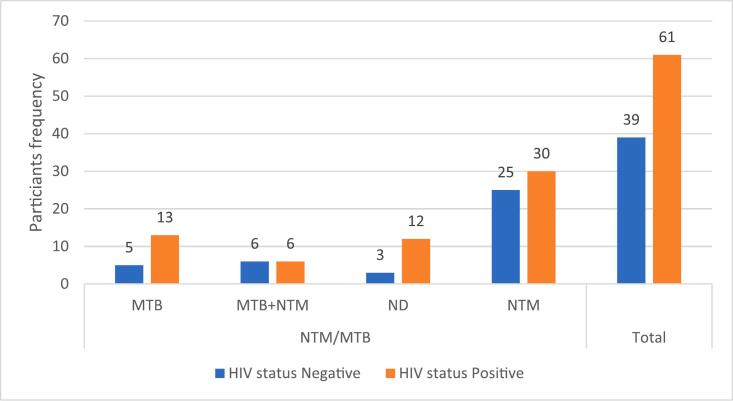
HIV co-infection associated with NTM/MTB co-occurrence.

Fig. 2B presents the prevalence rate of MTB and NTM among HIV-positive participants, where 63.33 % (19/30) cases of MTB were detected, and NTM cases were observed as more prevalent in these HIV-positive participants, 53.73 % (36/67).

## 4 Discussion

This cross-sectional study investigated bacterial co-occurrence and HIV infection in patients with pulmonary tuberculosis. The simultaneous presence of NTM (67 %), *Aeromonas spp* (19 %), *E. coli* strains (2 %), and *Vibrio spp* (2 %) in the analysed sputum samples showed a complex interplay. Polymicrobial infection is common in TB patients, particularly those with weakened immune systems and underlying conditions [26,27]. Notably, most samples (55 %) were negative for MTB, this could have been due to sputum samples were collected at random different stages of treatment of individual patients, some were new cases while others were already receiving treatment. some of the participants may have already been cured of the TB infection during the sample collection even though they were still yet to complete the treatment course. These findings could suggest the effectiveness of the anti-tuberculosis drugs administered in the health care facilities, as highlighted by Chakaya and colleagues [28], which reported a notable successful rate of treatment among TB patients who were receiving anti-TB treatment.

The majority (55 %) of participants were male, with a high TB prevalence observed in the patients aged between 36 and 55-year-old. According to the World Health Organization [29], males are generally more susceptible to TB infection than females especially male who work in conditions like mines, construction etc. Interestingly, the current study found that young females were more frequently affected by TB, while TB prevalence in males increased steadily from middle age through to older age. These findings are consistent with those of Patwardhan et al. [30], who reported higher global TB morbidity in men compared to women between the ages of 25 and 49, with an increase in the ages between 50 and 69-year group. The study area of Vhembe District is a rural setting where middle-aged men are often considered the primary breadwinners, which may give them more exposure than women and consequently get infected with tuberculosis.

The present study also found that male participants had the highest proportion of employment and self-employment (Table 2). This suggests that employment could influence social mixing patterns, with men having more frequent contact with individuals who may be TB-infected, thereby increasing their likelihood of exposure. A study conducted by Miller et al. [31] in Kampala, Uganda, similarly found that men recently diagnosed with TB had higher levels of contact with other men within their social networks. However, work-related risk factors and gender-specific behaviours are the main drivers of an increase in men's vulnerability to TB, which still requires further investigation. The findings did not suggest any significant association between gender or age and bacterial co-infection. Notably, *Vibrio* spp. and *E. coli* were evenly distributed across genders; however, their prevalence was insignificant. However, *Aeromonas* spp. was observed at a higher proportion in males (64.71 % of 17) cases; p-value = 0.85). Additionally, 53.73 % (36/67) of the NTM cases were found in male participants, but no significant association was observed with gender (p-value = 0.19) (Table 1).

In addition, a high prevalence of HIV co-infection (61 %) was observed, with male participants showing a slightly higher proportion of HIV-positive cases (61.82 %). A significant statistical association was found between age and HIV status, with a more significant proportion of participants aged 36–55 testing HIV-positive. This finding aligns with that of Tshitenge et al. [32], who reported a significant prevalence of TB-HIV co-infection in Botswana, where 54.7 % of cases involved individuals with both TB and HIV. TB and HIV co-infection are well-documented, as the two diseases often exacerbate each other's progression [33]. Although a high detection rate of bacterial species was noted in HIV-positive samples, no significant association (p = 0.22) was found between HIV status and the presence of bacterial co-infections (Fig. 2A). Additionally, 46.27 % (31/67) of NTM cases were found in HIV-positive participants, though no significant association was observed (p-value = 0.19) (Fig. 2B). Understanding a participant's HIV status is crucial in assessing their risk, as HIV is a known risk factor for the progression of latent TB infection to active TB disease [34]. Furthermore, the potential for TB outbreaks to spread rapidly within the HIV-positive population highlights the importance of addressing both infections simultaneously.

This study identified the presence of various bacterial species in sputum samples. A similar study conducted in Cambodia reported bacterial presence in 43.79 % of patients [35]. As immunity weakens during active TB, individuals with compromised immune systems are more susceptible to bacterial infections than healthier individuals [36]. *Aeromonas* species, ubiquitous in aquatic environments, can cause infections in immune-compromised patients, possibly by consuming hot water from storage tanks [21,37]. The present study detected Aeromonas species in 19 % of the TB-sputum samples, with a 31.58 % (6/19) detection rate in HIV-positive participants (Fig. 2A). While previously suspected to cause pneumonia, *Aeromonas* spp. is not commonly linked to respiratory infections [38]. However, there have been reports of *Aeromonas* spp. being associated with various infections, including those in the lower respiratory tract [39], and these cases have been reported in healthy individuals and those with underlying medical conditions [40].

The detection of *E. coli* (2 %) and *Vibrio* spp. (2 %) in the TB sputum samples in this study (Table 1), could be through contaminated food and water that people are exposed to in the rural setting [41]. *E. coli* detection in sputum is not uncommon [42], and Edwards et al. [43] reported a prevalence of 12.3 % of E. coli in sputum from patients with poor nutritional status and compromised lung function—pathogens such as *Aeromonas, E. coli, and Vibrio spp*. pose serious health risks, particularly to immune-compromised individuals [44]. Thus, the presence of species (*Aeromonas, E. coli, and Vibrio spp)* in the study participants may serve as a severe warning regarding the importance of proper hygiene, particularly for those immunocompromised, living with HIV, or diagnosed with TB.

Tuberculosis contributes to the threatening or weakening of patients' immune systems, such that co-infection in TB patients should be considered a deadly threat to life. Co-infection in TB patients has increased mortality and morbidity due to a lack of awareness during TB treatment. In this study, co-infection of MTB and NTM (12 %; 12/100) was noted with concern (Table 2), of which similar results were previously reported wherein detection of NTM was notably high in TB patients [45]. In a Korean study, He and colleagues [46] found that the proportion of NTM-TB co-infection among patients with NTM infection was 19.3 % (87/450). The detectable detection of NTM ultimately encourages further analysis and assessment of the possible risks associated with TB patients.

Studies have reported that NTM species are distributed depending on geographical regions, with the epidemiological infections varying between studies [47]. NTMs can be found in various environments that humans and animals share, such as water streams; these bacterial spp can infect humans by inhaling or ingesting them [48]. The zoonotic transmission of NTMs has also been suggested, especially among people close to animals, as most do in the Limpopo province, the study area. The current study was limited to detecting the presence of NTMs without the actual spp; thus, future studies should identify common NTMs found in the rural Vhembe district, where activities such as cattle herding are still common practice.

According to a WHO report (2017), issuing appropriate treatment combined with several quality-assured TB medicines reduces the risk of selecting resistant strains. From this study, only 2 % (2/100) of the participants undergoing TB treatment were identified to be drug-resistant. Thus, this could imply that most patients screened as TB positives adhered to the adequate and completed treatment prescribed by the health care workers. Although the drug resistance was minimal, the resistance to isoniazid and fluoroquinolones, among other drugs among the TB victims, have been reported with concern [49]. It has been suggested that MTB resistance strains can be transmitted to other persons through direct contact [50].

In contrast, preventative measures such as wearing face masks and hygiene practices can effectively prevent transmission of such TB-resistant strains. Previous studies have reported a reduction in the detection rate of MTB due to face masks by TB patients [51], which may suggest a positive role in TB spread prevention. However, an alarming number of participants did not adhere to such preventative measures in this study, as they also used public transportation for regular travel as they indicated.

## 5 Conclusion

This study emphasizes the challenge of TB among patients in rural communities in Vhembe. The findings suggest that age, socioeconomic status, and gender may play significant roles in the development of TB, HIV, and other bacterial infections, which could further complicate treatment outcomes. These factors contribute to increased vulnerability to co-infections, highlighting the complex interplay between TB and HIV in these populations. Additionally, the study emphasises the importance of considering these socio-demographic factors in public health interventions to reduce the burden of TB-HIV co-infection and associated bacterial infections. TB continues to pose a global health threat, making it essential to address the burden of *MTB* co-infection with other pathogens. Controlling these HIV and preventing TB is crucial for mitigating immunosuppression, co-infections and reducing mortality. This research contributes to a broader understanding of TB-related co-infections and their implications for treatment. Future studies on TB co-infections should be prioritised, particularly in rural areas, alongside enhanced public education on hygiene practices to prevent the transmission of co-infecting pathogens.

## Ethical approval

The study protocol underwent ethical review and approval by the University of Venda Research Ethics Committee (UREC) under the reference number SMNS/20/MBY/13/2104. Additional ethical clearance was obtained at the provincial level from the Limpopo Provincial Department of Health (LP_2021-11–001). The Vhembe District Department of Health granted permission to access healthcare facilities.

## CRediT authorship contribution statement

**Mpho Magwalivha:** Writing – review & editing, Writing – original draft, Methodology, Investigation, Formal analysis, Data curation, Conceptualization. **Mpumelelo Casper Rikhotso:** Writing – review & editing, Writing – original draft, Investigation, Formal analysis, Data curation, Conceptualization. **Leonard Owino Kachienga:** Writing – original draft. **Rendani Musoliwa:** Writing – review & editing, Writing – original draft, Investigation, Formal analysis, Conceptualization, Methodology, Software, Visualization. **Ntshunxeko Thelma Banda:** Writing – review & editing, Data curation, Conceptualization. **Maphepele Sara Mashilo:** Writing – review & editing. **Thembani Tshiteme:** Writing – review & editing, Methodology, Investigation, Data curation, Conceptualization. **Avheani Marry Mphaphuli:** Writing – review & editing, Methodology, Investigation, Formal analysis, Data curation. **Hafsa Ali Mahamud:** Writing – review & editing, Methodology, Investigation, Formal analysis, Data curation. **Sana Patel:** Writing – original draft, Methodology, Investigation, Formal analysis, Data curation. **Jean-Pierre Kabue Ngandu:** Writing – review & editing, Methodology, Investigation, Formal analysis, Data curation. **Natasha Potgieter:** Writing – review & editing, Supervision, Resources, Project administration, Methodology, Funding acquisition, Conceptualization. **Afsatou Ndama Traoré:** Writing – review & editing, Writing – original draft, Supervision, Resources, Project administration, Methodology, Investigation, Funding acquisition, Formal analysis, Data curation, Conceptualization.

## Funding

Not applicable.

## Declaration of competing interest

The authors declare that they have no known competing financial interests or personal relationships that could have appeared to influence the work reported in this paper.
